# Characterization and Expression Analysis of the SABATH Gene Family Under Abiotic Stresses in Cucumber (*Cucumis sativus* L.)

**DOI:** 10.3390/plants14121748

**Published:** 2025-06-07

**Authors:** Xinjie Zhang, Shanyu Li, Yang Zhou, Mengxin Chen, Lisi Jiang, Wei Fu

**Affiliations:** College of Life Science, Shenyang Normal University, Shenyang 110034, China; 15041158087@163.com (X.Z.); 15524253653@163.com (S.L.); zy1925816377@163.com (Y.Z.); mxchen0110@163.com (M.C.)

**Keywords:** cucumber, SABATH gene family, drought and salt stress

## Abstract

SABATH methyltransferase can methylate small-molecule metabolites of plants to generate different products, and it plays a crucial role in plant growth and development as well as stress response. In this study, 13 SABATH genes distributed on five chromosomes of cucumbers were identified, and the synergistic effects among their domains, gene structures, conserved motifs, phylogenetic relationships, collinearity analysis, cis-acting elements, expression patterns, and plant growth-promoting rhizosphere bacteria (PGPR) were analyzed. The gene structure and conserved motifs of the same group of CsSABATH have similar intron numbers and conserved motifs. We detected 10 cis-elements in the promoter of the CsSABATH gene, indicating that they may be involved in different signaling pathways. qRT-PCR revealed the tissue-specific, drought and salt stress-responsive expression of the SABATH gene in cucumbers. Furthermore, we also verified that the expression level of *CsaV3_6G046510* after inoculation with PGPR-GD17 bacteria under drought and salt stress was significantly lower than normal drought and salt dress, indicating that this gene may respond to PGPR and in abiotic stress play an important role. This study provides valuable insights into the molecular characteristics and evolutionary history of the SABATH gene family in cucumbers, laying a foundation for further analysis of the function of the CsSABATH gene in cucumbers.

## 1. Introduction

SABATH methyltransferase is an enzyme that catalyzes the methylation of plant hormones and other small molecules by recognizing carboxyl O atoms or amino N atoms in small molecules [1]. The resulting products participate in the biosynthesis of various secondary metabolites and play a crucial role in plant growth, development, and defense. The enzymes are named after the first three enzymes: SA is salicylic acid carboxyl methyl-transferases (SAMT); BA is benzoic acid carboxyl methyltransferases (BAMT); and TH is theobromine synthase [2]. So far, SABATH methyltransferases have been identified in a variety of species, such as *Arabidopsis thaliana* (24), *Oryza sativa* L.(21), *Solanum lycopersicum* L. (20), and others [3,4].

The *SABATH* genes are extensive in plants, ranging from algae to higher plants. And the number and function of its family members have also expanded [5]. As mentioned earlier, SABATH methyltransferases have many catalytic substrates, and a given gene in this family can also catalyze different substrates, indicating the diversity of gene function. For example, 24 genes have been identified in *Arabidopsis thaliana* [3], among which *AtJMT* is involved in plant defense responses [6], *AtIAMT* regulates plant development and maintains auxin homeostasis [7], *AtGAMT1* and *AtGAMT2* promote seed development [8], *AtPXMT1* promotes seed germination [9], and *AtNAMT1* plays a role in abiotic stress [10]. In addition, in rice, *OsJMT* regulates rice growth and development and participates in plant defense response [11]; *OsIAMT1* functions similarly to *AtIAMT1*, participating in rice development and controlling auxin homeostasis [12]. In *S. lycopersicum*, both *SlSAMT* and *SlJMT* are directly or indirectly involved in plant defense responses [13,14].

At present, many reports have confirmed that plant growth-promoting rhizosphere bacteria (PGPR) can cope with the damage caused by abiotic stress by regulating different mechanisms, help plants survive, and improve the adaptability of plants to adverse conditions, making them capable of normal development and reproduction [15,16,17,18]. In our previous research, it was proven that GD17 can act as PGPR to promote the growth of rice and improve its salt resistance [19]. Meanwhile, the inoculation of Chinese cabbage with PGPR-GD17 can increase the resistance of Chinese cabbage to cadmium in multiple ways, such as reducing abscisic acid (ABA) content and enhancing photosynthesis under cadmium stress [20]. Therefore, this study explored the expression of the *SABATH* family in cucumbers under the conditions of PGPR-GD17 inoculation and uninoculation in order to further verify the role of this family.

Cucumber (*Cucumis sativus* L.), belonging to the Cucurbitaceae family, is one of the most economically important vegetable crop varieties. Members of the *SABATH* gene family have been widely studied in *Arabidopsis thaliana*, but rarely in cucumbers. Therefore, it is of great significance to identify the *SABATH* gene family of cucumbers and analyze its expression under stress. In this study, 13 *SABATH* genes were identified at the whole-genome level of cucumbers using bioinformatics methods. Meanwhile, a systematic analysis was conducted on its physical and chemical properties, chromosome position, conserved motifs, gene structure, phylogenetic analysis, collinear analysis, cis-acting elements, expression levels of tissues (roots, leaves, male flowers, female flowers, small fruits, tendrils), the expression of each gene under stress, and how it participates in the role of PGPR on abiotic stress in cucumbers. This study lays a foundation for in-depth research on the biological functions and evolutionary processes of the *SABATH* family genes in cucumbers.

## 2. Results

### 2.1. Identification and Characterization of CsSABATH Gene Family

**Figure 1:**
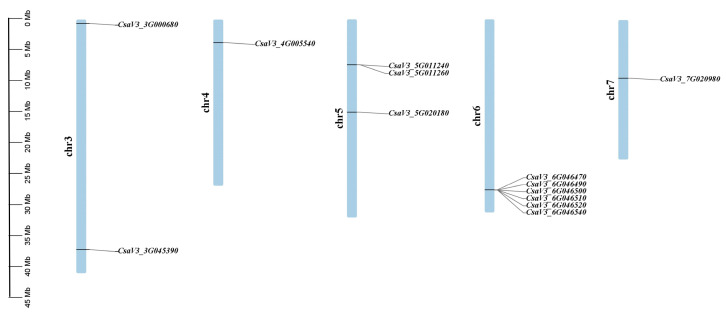
Chromosomal distribution and localization of *CsSABATHs*. The chromosome names are shown on the left of each chromosome. The chromosome scale is in millions of bases (Mb) on the left.

A total of 13 *SABATH* family members using HMMER 3.0 software from the cucumber genome were identified. The existence of the conserved SABATH domain was verified by Pfam and SMART database. These 13 *CsSABATH* genes were distributed on five different chromosomes of cucumber (Figure 1). Only one gene was present on chromosomes 4 and 7, while chromosome 3 had two genes. There were more *SABATH* genes on chromosomes 5 and 6. Chromosome 5 included three genes and chromosome 6 had the highest number of genes—six genes. Both *CsaV3_5G011240* and *CsaV3_5G011260* were located on the same site of Chr 5 and belonged to a pair of tandem duplication genes. In addition, the six genes located on chromosome 6 were also in the same location and were tandem duplication genes [21].

The physical and chemical properties of 13 cucumber *SABATH* genes and encoded proteins, including location, protein length, molecular weights, protein isoelectric points (pI), instability indexes, grand average of hydropathicity (GRAVY) values, and subcellular localization, were analyzed as shown (Table 1). The number of amino acids of CsSABATH proteins ranged from 295 to 459 aa. The molecular weights of 13 CsSABATH proteins were distributed between approximately 33.4 and 51.9 kDa. The pI of 13 CsSABATH proteins varied from 5.36 (*CsaV3_6G046520*) to 8.84 (*CsaV3_6G046540*). Except for *CsaV3_6G046490* and *CsaV3_6G046500*, the instability index was greater than 40 in all CsSABATH proteins, which advised that most CsSABATH proteins were unstable proteins, except *CsaV3_6G046490* and *CsaV3_6G046500*. The GRAVY values was less than zero for each CsSABATH protein, except *CsaV3_5G020180*, indicating that these proteins were hydrophilic. Subcellular localization prediction revealed that all *CsSABATH* genes were located in the cytoplasm or outer membrane. It is worth noting that the physicochemical properties of the tandem duplication genes mentioned above in Table 1 are not similar. We speculate that this might be because the copy number of genes or alleles varies during tandem duplication, resulting in different physical and chemical properties and the possible different functions they perform [22].

### 2.2. The Conserved Motifs and Gene Structure of CsSABATH Genes

**Figure 2:**
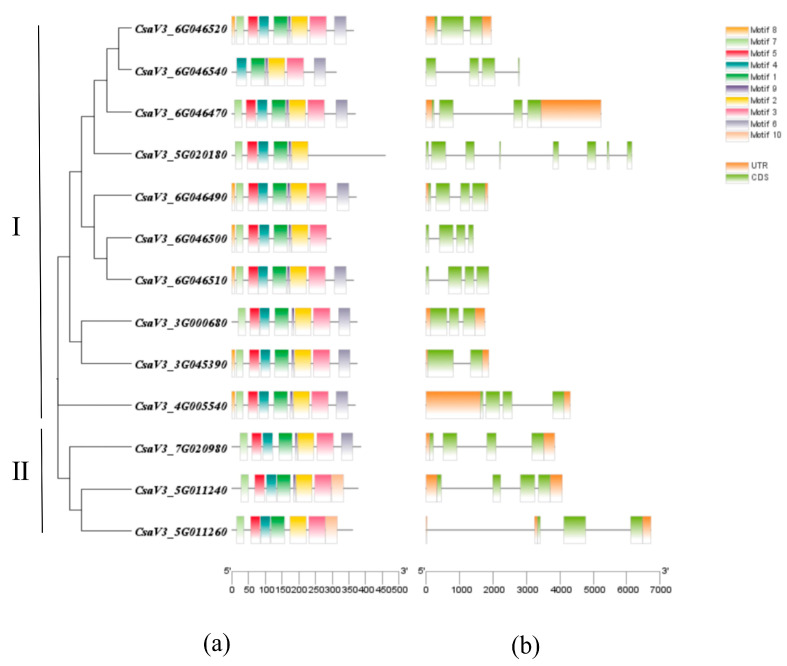
Phylogenetic relationships, gene structure, and conserved motifs of *CsSABATH* genes. (**a**) The phylogenetic relationship of SABATH within cucumbers themselves. Tree was constructed by the maximum likelihood method and distributions of conserved motifs in SABATH proteins. Motifs are indicated by 10 different colored boxes. (**b**) Exon/intron architectures of *SABATHs*. Green-colored boxes indicate exons, and the plain line indicates introns.

To investigate the evolutionary relationship of *CsSABATHs* in cucumber, we created an unrooted phylogenetic tree by aligning the 13 CsSABATH protein sequences. The conserved motifs and gene structures of the *CsSABATH* genes were researched based on their phylogenetic relationships (Figure 2). *CsSABATHs* could be clustered into two groups (I, II) (Figure 2a). A total of 10 conserved motifs were identified in the 13 CsSABATH proteins, designated as motifs 1 to 10. The amino acid sequences of the conserved motif are shown in Supplementary Table S1. Most CsSABATH proteins generally contained similar conserved motif compositions in the same group (Figure 2a). For example, the CsSABATH proteins in group I included motifs 1, 2, 4, and 9, and those in group II included motifs 1, 2, 3, 4, 5, and 7. Among them, motifs 1, 2, and 4 were present in all CsSABATH proteins, and we speculated that motifs 1, 2, and 4 were highly conserved during cucumber development. In addition, the conserved motif compositions of CsSABATH proteins were similar in the same group but varied among different groups. According to the comparison results, the exons of CsSABATHs are mostly four. Furthermore, *CsaV3_3G045390* has two exons; *CsaV3_6G046520*, *CsaV3_3G000680*, and *CsaV3_5G011260* all have three; and *CsaV3_5G020180* has eight. This is consistent with the results of *Arabidopsis* and rice. Further analysis showed that the CsSABATH proteins in group I mostly had two or three introns, but they also had one and seven introns, while the CsSABATH proteins in group II had two or three introns (Figure 2b). In short, the gene structure and highly conserved motif of *CsSABATH* further support the phylogenetic analysis’s reliability and close evolutionary relationship.

### 2.3. Phylogenetic Tree of CsSABATH Genes

**Figure 3:**
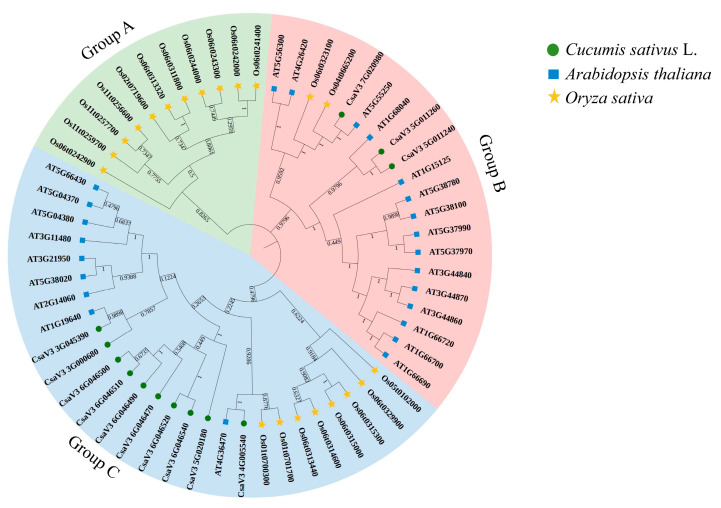
SABATH phylogenetic trees of three plants divided into three subgroups. *SABATH* members of cucumber, *Arabidopsis*, and rice are represented by a green circle, blue rectangle, and yellow five-pointed star, respectively. The number on each branch represents the percentage of self-expanding value.

To analyze the phylogenetic relationship of the *CsSABATH* genes among different species and classify the *CsSABATH* genes, we constructed a maximum likelihood phylogenetic tree based on the multiple-sequence alignment of 13 cucumber SABATH proteins, 21 *Arabidopsis thaliana* SABATH proteins, and 24 *Oryza sativa* SABATH proteins (Figure 3). We divided the *SABATH* genes into three groups according to their phylogenetic relationship, named Group A to Group C. The 13 *CsSABATH* genes were distributed in Group B and Group C, among which 3 *CsSABATH* genes were in Group B and 10 *CsSABATH* genes were in Group C. This is consistent with the phylogenetic analysis of cucumber itself. In Group A, only the *SABATH* gene in rice was present, which might be due to the distant homologous relationship between rice and cucumber. At the same time, most of the identified *SABATH* gene family in cucumber comprised paralogs genes, such as *CsaV3_5G011240* and *CsaV3_5G011260*, *CsaV3_6G046500*, and *CsaV3_6G046510*. Moreover, phylogenetic trees showed that cucumber and *Arabidopsis* are more closely related to each other than cucumber and rice. This phylogenetic difference may be the result of multiple factors, including functional differentiation, environmental adaptation, and so on. These differences highlighted the complexity of plant evolution and the complex ways plants adapted to their environment over time. All in all, phylogenetic analysis could provide valuable insights into the evolution and function of family genes.

### 2.4. Collinearity Analysis of CsSABATH Family Genes

**Figure 4:**
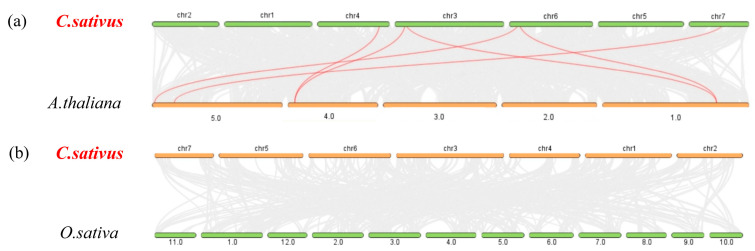
The collinear relationship between the *SABATH* gene family of cucumbers and other species. The red lines represent gene pairs. The collinear relationship of all direct homologous genes in different species is represented by gray lines. (**a**) Collinearity of the direct homologous *SABATH* gene in *Arabidopsis thaliana* and cucumber. (**b**) Collinearity of the direct homologous *SABATH* gene in rice and cucumber.

We jointly analyzed *Arabidopsis SABATH*, rice *SABATH*, and cucumber *SABATH* genomes to study their collinear genetic relationships. The collinearity analysis showed that there were six pairs of collinear genes between the cucumber and *Arabidopsis* genomes (Figure 4). Among them, only the *CsaV3_7G020980* gene in group II and three genes in group I had a relationship with *Arabidopsis SABATH* [3]. However, there were zero pairs of collinear genes between the cucumber and rice genomes (Figure 4). These results suggest that these genes may have functionally differentiated during the evolution of cucumber and *Arabidopsis*. We hypothesize that functional differentiation of these genes may have occurred during the evolution of cucumber and *Arabidopsis*.

### 2.5. Analysis of Cis-Acting Elements of CsSABATH Promoter

**Figure 5:**
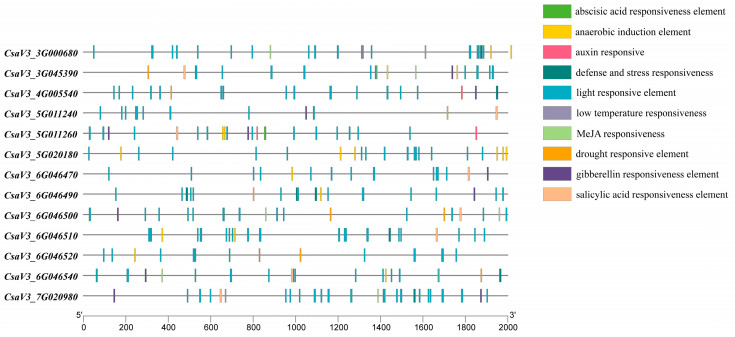
Cis-element analysis in the promoters of *CsSABATH* genes. The above figure shows the position of cis-elements at 2 kb upstream of *CsSABATH* gene. Different colored squares represent different elements.

The 2000bp sequence before the start codon is selected to predict the cis-acting element. As shown in Figure 5, we divided the regulatory elements of cucumber *SABATH* into three categories, namely light-responsive elements, hormone-responsive elements, and pressure-responsive elements. Among them, the optical response element is the largest component group. Each *CsSABATH* promoter includes a large number of light-responsive elements, which means that *CsSABATH* may be differentially regulated by light. Cis-regulatory elements involved in abiotic stress were also detected in a range of members, such as drought response elements, defense and stress response elements, and anaerobic induction elements. In addition, five types of hormone response elements were identified, and all *CsSABATH* promoter sequences contained at least one element involved in plant hormone response, except *CsaV3_5G020180*, including abscisic acid (ABA)-responsive element (ABRE), methyl jasmonate (MeJA)-responsive element (CGTCA-motif and TGACG-motif), gibberellin-responsive elements (GARE-motif and P-box), salicylic acid-responsive elements (TCA-element), and auxin-responsive elements (TGA-element and AuxRR-core). There are many cis-elements related to light, stress, or hormone response in the promoter of *CsSABATH*, indicating that *CsSABATHs* are related to various stress and hormone responses.

### 2.6. Tissue-Specific Expression Profiles of CsSABATH Genes

**Figure 6:**
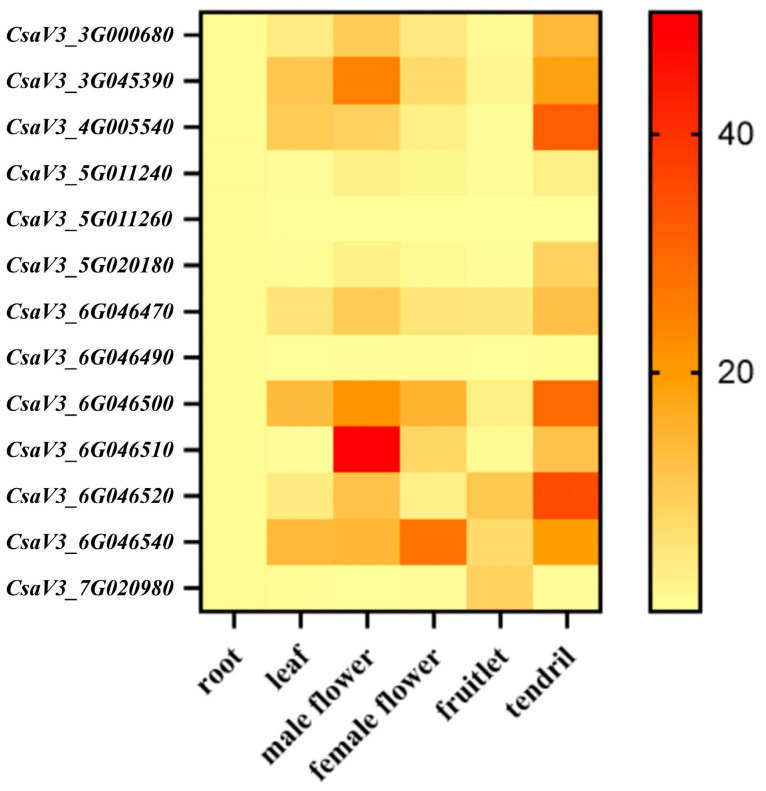
Expression patterns of *CsSABATH* genes in various cucumber tissues. The expression values mapped to a color gradient from low (yellow) to high expression (red) are shown at the right of the figure.

To better understand the role of *CsSABATH* genes in the growth and development of cucumber, the expression patterns of 13 *CsSABATH* genes in six different tissues were analyzed by qRT-PCR (Figure 6). The results showed that all *CsSABATH* members were lowly expressed in the root tissue. In addition, only *CsaV3_5G011260* and *CsaV3_6G046490* were under-expressed in all tissues of the cucumber *SABATH* family members. The expression of *CsaV3_7G020980* was high in fruit and low in other tissues. In addition, the expression levels of all genes were higher in male flowers and tendrils. The expression of *CsaV3_6G046510* was the highest in male flowers. Interestingly, only the expression levels of *CsaV3_3G045390* and *CsaV3_6G046510* in male flowers were higher than those in other tissues. Therefore, we speculate that *CsaV3_3G045390* and *CsaV3_6G046510* might be related to the development of male flowers.

### 2.7. Relative Expression of the CsSABATH Gene Under Osmotic Stress Treatment

**Figure 7:**
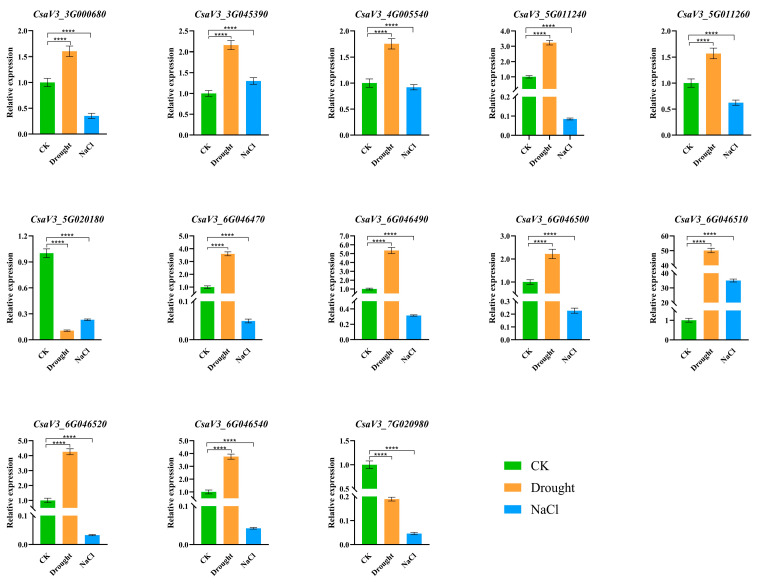
Expression profiles of *CsSABATH* genes in response to various stress. Green represents normal cucumbers, orange represents cucumbers treated after 9 days of drought, and blue represents cucumbers treated with 100 mM of NaCl. The relative expression levels of *CsSABATH* genes were analyzed at stress as compared with their values at normal. The gene relative expression was calculated using the 2^−ΔΔCt^ method with CsActin as an internal control, and the value represents the mean ±SE of three biological replicates. Asterisks indicated values that are significantly different from CK (**** *p* < 0.0001, one-way ANOVA).

To determine whether *CsSABATH* is involved in osmotic stress response, quantitative reverse transcription–polymerase chain reaction (qRT-PCR) was used to analyze their expression levels under drought and salt treatment (Figure 7). In the drought treatment group, compared with the control group, only *CsaV3_5G020180* and *CsaV3_7G020980* were lower expression. Furthermore, *CsaV3_6G046510* was highly expressed up to 35 times. In addition to the above three genes, the expression of the remaining 10 genes ranged from 1.5 to 5 times higher. In the salt treatment group, the expression of most genes was lower than that of the control group. It is worth mentioning that *CsaV3_6G046510* is highly expressed up to 50 times. In summary, the expression patterns of the two groups indicate that *CsaV3_6G046510* may play an important role in osmotic stress. These results provide a basis for future functional studies of *CsSABATH* gene.

### 2.8. The Functional Prediction of CsaV3_6G046510 and PGPR-GD17 Can Alleviate the Response of CsaV3_6G046510 to Abiotic Stress

**Figure 8:**
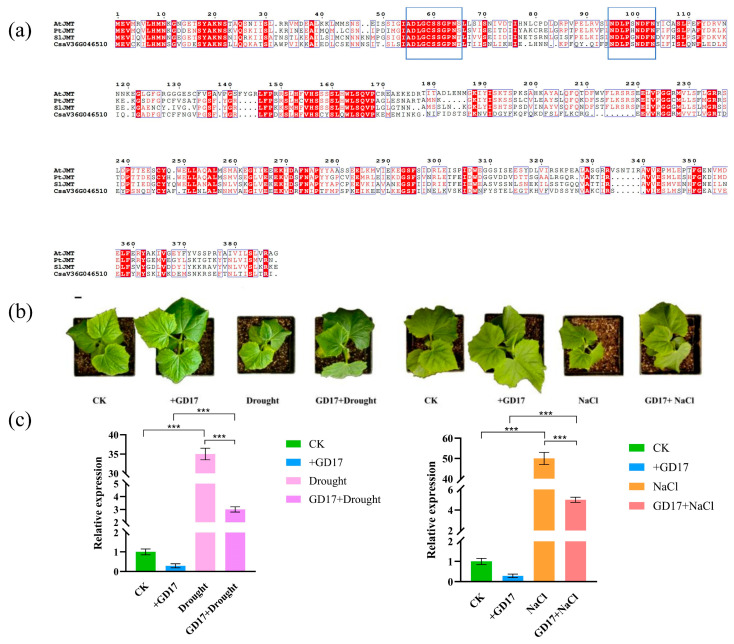
(**a**) Comparison the *CsaV3_6G046510* amino acid sequence with *AtJMT*, *PtJMT*, and *SlJMT* amino acid sequence. Boxes represent amino acid residues that are identical among the four proteins. (**b**) Cucumber seedlings inoculated with PGPR-GD17 and/or treated with drought and salt stress. Bar = 1 cm. (**c**) Relative expression of *CsaV3_6G046510* gene. The *** on the column indicates the significance of the difference compared with the control (*p* <  0.001).

Based on the specific expression of *CsaV3_6G046510* in male flower and its high expression under drought and salt stress, we conducted a further analysis of *CsaV3_6G046510*. It was found that among the 24 AtSABATHs, the protein sequence of *CsaV3_6G046510* had the highest identity with the *AtJMT* gene. We compared the protein sequences of the *JMT* genes that have been identified so far (Figure 8a). It was found that *CsaV3_6G046510* had a relatively high similarity with *JMT* protein sequences in *Arabidopsis thaliana* [6], poplar [23], and tomato [24]. Therefore, we speculate that *CsaV3_6G046510* might be *JMT*.

To continue exploring the expression of *SABATH* members in cucumbers under drought and salt treatment, we also conducted qRT-PCR analysis on cucumbers inoculated with GD17 bacteria under drought and salt treatment conditions (Figure 8b,c). Based on the tissue expression pattern and the qRT-PCR results of abiotic stress, *CsaV3_6G046510* was selected as the gene for subsequent research. The results showed that whether under drought stress or salt stress, the expression level of *CsaV3_6G046510* in cucumbers inoculated with GD17 was dozens of times lower than that in cucumbers treated under normal drought and salt stress (Figure 8b,c). This indicates that *CsaV3_6G046510* may be involved in abiotic stress mediated by GD17.

## 3. Discussion

The SABATH methyltransferase family is a family of proteins that catalyze the methylation of carboxylic acids to form methyl esters [1]. The substrates of the *SABATH* family are extensive, including benzoic acid, gibberellic acid, cinnamate/*p*-coumarate, and farnesoic acid [5,8,25,26]. The products of these small-molecule reactants, the corresponding methyl esters, may have different biological functions than their substrates [27]. Plant hormones are one of the important regulatory factors for plant growth, development, and stress response [28]. At present, methyltransferases with plant hormones as substrates, such as *SAMT, JMT, IAMT*, etc., have been studied more.

### 3.1. Tandem Duplication Might Be One of the Reasons for the Diverse Functions of the CsSABATH Family

Tandem duplication is composed of identical sequences in close genomic proximity and occurs due to unequal chromosomal crossing over [29]. Tandem duplication occurs more frequently in the plant genome compared to other duplication patterns. From the existing results (Figure 1), *CsaV3_5G011240* and *CsaV3_5G011260* at the same site on chromosome 5 and *CsaV3_6G046470*, *CsaV3_6G046490*, *CsaV3_6G046500*, *CsaV3_6G046510*, *CsaV3_6G046520*, and *CsaV3_6G046540* at the same site on chromosome 6; that is, most genes in *CsSABATH* may suggest potential tandem duplication events [30]. For the *SABATH* family in other species [3,31,32], tandem duplication for gene expansion also exists, which may be the main expansion mechanism of this gene family. Meanwhile, the expression of these tandem genes in cucumber tissues was different (Figure 6). The expression of CsSABATH genes in root was all low. Although the expression levels of *CsaV3_5G011240* and *CsaV3_6G046510* in flowers and tendrils were higher than other tissues, the overall expression levels were relatively low. The expression levels of *CsaV3_5G011260* and *CsaV3_6G046490* were almost all low. In addition, these genes also showed different expression trends in response to different stresses (Figure 7). In particular, *CsaV3_6G046510* was significantly upregulated. In salt stress, it was mainly downregulated; only *CsaV3_3G045390* was slightly upregulated, and *CsaV3_6G046510* was significantly upregulated, up to more than 50 times. The expression of *CsSABATH* genes in a variety of cucumber tissues is consistent with previous studies of the broad biological functions of the *SABATH* family [3,33]. Moreover, differences in expression between members of the same locus of *CsSABATH* genes indicate possible functional differentiation between them, indicating the functional complexity of the *SABATH* family in cucumber. At the same time, the expression of *CsSABATH* genes in response to different stresses is also different, which may confirm the different and extensive biological functions of the *SABATH* family.

### 3.2. Analysis of Phylogenetic Relationships, Gene Structures, and Cis-Acting Elements

Phylogenetic analysis showed that only rice was found in Group A, while cucumber was found in Group B and Group C. There are 3 cucumber genes in Group B and 10 in Group C (Figure 3), which are consistent with the results of the phylogenetic analysis of cucumber itself (Figure 2a). In phylogenetic tree, *CsaV3_3G045390* and *AT1G19640* are one branch; *CsaV3_4G005540* and *AT4G36470* are one branch; and *CsaV3_7G020980* and *AT5G55250* are one branch, indicating that they are closely related to each other. The results were consistent with the collinearity analysis of cucumber–*Arabidopsis* (Figure 4a). The above results show that the functions of *CsaV3_3G045390* and *AT1G19640*, *CsaV3_4G005540* and *AT4G36470*, and *CsaV3_7G020980* and *AT5G55250* may be similar. In addition, the tandem replication gene mentioned earlier is also one branch. In addition, in Group A, only rice is included. We speculate that this category might have only been obtained when separated from the last common ancestor [34]. This hypothesis needs to be further confirmed.

Then, we combined the phylogenetic analysis of cucumbers with conserved motifs analysis and gene structure analysis and found that the members of group I contained conserved motifs 1, 2, 4, and 9, and most of them contained 2–3 introns. All members of group II contained conserved motifs 1, 2, 3, 4, 5, and 7 and 2–3 introns. The arrangement of exons and introns within genes serves as a crucial indicator of the evolutionary connections among gene family members [35]. The result suggests that the genes of the same group have similar conserved motifs and gene structures. Furthermore, the conserved motif 8 only appeared in group I, and the conserved motif 10 only appeared in group II, indicating that the *CsSABATH* gene may have structural differences in different groups.

Among the cis-acting elements, we found that only *CsaV3_5G020180* did not contain hormone-related elements, while the other genes all contained hormones and stress elements. *CsaV3_5G011260* contains four different hormone components and is the gene with the most hormone components. *CsaV3_3G045390*, *CsaV3_6G046500*, and *CsaV3_6G046540* contain three hormone elements, while most of the remaining genes contain only one hormone element. *CsaV3_3G000680*, *CsaV3_6G046490*, *CsaV3_6G046500*, and *CsaV3_6G046540* contain three stress-related components. Moreover, the expression levels of *CsaV3_6G046490*, *CsaV3_6G046500*, and *CsaV3_6G046540* under drought stress were higher compared with other genes (Figure 7). Five genes contain two stress-related elements, and five genes contain one stress-related element. Compared with other genes, *CsaV3_6G046500* and *CsaV3_6G046540* contain more hormone elements and more stress-related elements (Figure 5).

### 3.3. Functional Speculation of CsaV3_6G046510

Due to the extremely high expression of *CsaV3_6G046510* in male flowers and abiotic stress, we subsequently speculated its function. It has been confirmed in previous hormone studies that the jasmonic acid (JA) pathway is crucial for the development of stamens in *Arabidopsis thaliana* [36]. Based on this, we hypothesize that the substrate of *CsaV3_6G046510* might be JA. JA is a plant signaling molecule that enhances resistance in plants under abiotic stress by participating in physiological and molecular responses [37]. Meanwhile, JA and its various multiple conjugates, such as methyl jasmonate (MeJA), derivatives jasmonoyl-isoleucine (JA-Ile), etc., jointly constitute jasmonate (JAs) [38]. The exogenous application of JA or MeJA can alleviate oxidative damage in plants caused by drought stress [38,39,40]. Furthermore, JAs can increase antioxidant reactions to alleviate plant salt stress [41,42]. Studies on various plants have proved that JA synthetic genes are involved in drought resistance [43]. *CsaV3_6G046510* was upregulated by tens of times in both drought and salt stress (Figure 7). At the same time, studies have shown that *AtJMT* is specifically expressed in flowers [6]. After comparison, it was found that the *AtJMT* protein in *CsaV3_6G046510* had the highest identity with 24 AtSABATHs. Meanwhile, the protein sequences of *AtJMT*, *PtJMT*, *SlJMT*, and *CsaV3_6G046510* were compared; they all had common amino acid sites. These results suggest that *CsaV3_6G046510* might be *JMT*. However, the functional verification of genes is a task that requires complex and rigorous experiments for confirmation. We will further verify the function of *CsaV3_6G046510* in subsequent research.

## 4. Materials and Methods

### 4.1. Plant Culture and Treatment

Cucumber (*Cucumis sativus* L. cv., Zhongnong 26) seeds were obtained from the Institute of Vegetable and Flower Research, Chinese Academy of Agricultural Sciences. The seeds were sterilized with 75% anhydrous ethanol and germinated in a dark environment at 25 °C. After a successful 48 h, they were transplanted into seedling pots. The environment was set with a culture condition of 26 °C/18 °C, a photoperiod of 14/10 h, and a light exposure of 12,000 lx. The ratio of soil is soil/vermiculite/perlite = 3:2:1.

After 7 days of cultivation, cucumber seedlings demonstrating comparable developmental stages were selected for the experiment and divided into 2 groups (group CK and group +GD17). When the cucumbers began to grow their first true leaves, we centrifuged the GD17 bacterial solution that had been cultivated in a shaker for 48 h and then pipetted it evenly in pure water to make a 10^8^ concentration bacterial suspension, which was added to the cucumber seedlings. Seven days later, we added the bacteria again, as described above, and then cultivated it under normal conditions. At this moment, cucumbers with the same growth were selected and divided into 6 groups (group CK, group drought, group NaCl, group +GD17, group GD17 + drought, and group GD17 + NaCl). Cucumbers should be processed when they have grown to four true leaves. The drought treatment group was deprived of water for 9 days. The salt treatment group was treated with 100 mmol/L of salt treatment once every 3 days a total of twice. The above three groups all took the third true leaf of cucumber as the sample.

### 4.2. Identification of SABATH Genes in Cucumber

In order to identify candidate SABATH genes in cucumber, 21 SABATH protein sequences of *Arabidopsis* were used to BLASTp in the cucumber Chinese Long v3 database (http://cucurbitgenomics.org/ (accessed on 18 February 2025)) online, set as e-value < 1 × 10^−10^. We deleted any redundant results manually. The SABATH domain’s HMM profile (PF03492) was initially acquired from Pfam (http://pfam.xfam.org/ (accessed on 18 February 2025)) [44]. Using this computational model, the cucumber genome dataset underwent HMMER 3.0-based homology searches. Putative genes were subsequently selected via TBtools 2.0 [45] screening, followed by secondary validation using domain analysis tools from Pfam (http://pfam.xfam.org/search#tabview=tab1 (accessed on 19 February 2025)) and SMART databases.

### 4.3. Chromosome Distribution Bioinformatic Analyses of SABATH Gene Family in Cucumber

For chromosome distribution, the gff3 file of Chinese long V3 was downloaded from the Cucurbitaceae genome database. For chromosomal gene mapping and distribution analysis, TBtools was utilized. Protein sequence characterization, including physicochemical parameter prediction, was performed using the ProtParam online tool (http://web.expasy.org/protparam/ (accessed on 20 February 2025)). The subcellular compartmentalization of proteins was subsequently determined through the CELLO v2.5 platform (http://cello.life.nctu.edu.tw/ (accessed on 20 February 2025)).

### 4.4. Conserved Motif and Gene Structure Analysis

The phylogenetic tree of the cucumber SABATH gene family was constructed using the maximum adjacency method in MEGA11 software (accessed on 1 March 2025). Next, the online tool MEME (http://meme-suite.org/tools/meme (accessed on 1 March 2025)) was used to analyze the conserved sequence of cucumber SABATH, default to other parameters, and modify the maximum basis number to 10. Gene Structure View in TBtools and gff3 file of Chinese long V3 were used to generate the gene structure map. Finally, TBtools was used for the visual analysis.

### 4.5. Construction of Phylogenetic Tree

The evolutionary relationships among the SABATH gene family members in cucumber, *Arabidopsis*, and rice were analyzed through phylogenetic reconstruction. Using MEGA 11’s Muscle algorithm for multiple-sequence alignment, we generated a minimum-evolution tree to represent their genetic divergence. The resultant phylogenetic tree was subsequently visualized and optimized through the iTOL (https://itol.embl.de/ (accessed on 2 March 2025)).

### 4.6. Detection of Homologous Gene Pairs and Synteny Analysis

The MCScanX software of TBtools 2.0 (Multiple Collinearity Scan toolkit) was employed with default parameters to detect homologous gene pairs and relationships within the cucumber SABATH gene family. To infer the potential functions of cucumber SABATH genes, the synteny relationships of orthologous SABATH genes between cucumber and model organisms (*Arabidopsis* and rice) were analyzed. Additionally, the synteny connections of SABATH genes among cucumber, *Arabidopsis*, and rice were investigated using MCScanX under default settings. TBtools was utilized to create a homologous analysis chart illustrating the homologous association SABATH genes across cucumber, *Arabidopsis*, and rice.

### 4.7. Analysis of Cis-Acting Elements in SABATH Gene Promoters

A 2000 bp region upstream of the start codon of CsSABATH family genes was intercepted by TBtools as a promoter. Subsequently, the PlantCare website predicted the cis-acting elements (http://bioinformatics.psb.ugent.be/webtools/plantcare/html/ (accessed on 3 March 2025)).

### 4.8. Promoter Cis-Element Analysis

The 2000bp upstream genome sequences of the 13 cucumber SABATH gene start codon (ATG) were extracted by TBtools. The above sequences were then submitted to the online site plant care (http://bioinformatics.psb.ugent.be/webtools/plantcare/html/ (accessed on 3 March 2025)) for prediction. Finally, TBtools was used for the visual analysis.

### 4.9. RNA Isolation, cDNA Synthesis, and Quantitative Real-Time PCR Analysis

RNA extraction was performed with the total RNA extraction kit (Promega, Madison, WI, USA), and cDNA generation was conducted using the PrimeScript RT reagent set (TaKaRa, Kusatsu, Japan) in strict accordance with the standardized protocols. The subsequent quantification of gene expression levels was carried out on a Lightcycler 96 RT-qPCR platform (Roche, Basel, Switzerland). The gene expression level was analyzed according to the amplification conditions. Using the amplification conditions as the internal control, the relative expression of each gene was calculated by the 2^−ΔΔCT^ method. Subsequently, CK was set as 1, and the expression levels of the other groups were compared with CK. The sequence of gene-specific primers is shown in Supplementary Table S2.

### 4.10. Identity Comparison

We uploaded the 24 SABATH protein sequences of *Arabidopsis thaliana* and the *CsaV3_6G046510* protein sequence of cucumber to the CLUSTALW website for comparison.

## 5. Conclusions

In this study, thirteen SABATH genes were identified in cucumbers, distributed on 5 chromosomes, among which only one gene was located on chromosomes 4 and 7. These 13 CsSABATH genes have conserved motifs. Furthermore, the qRT-PCR results showed that CsSABATH was involved in the plant’s response to osmotic stress, mainly upregulated under drought stress and downregulated under salt stress. It is worth mentioning that *CsaV3_6G046510* showed significant responses under both treatments, and its expression level decreased due to the relief of the abiotic stress degree by vaccination with PGPR-GD17. Therefore, this gene may play an important role in the abiotic stress of cucumbers. This study provides a basis for the functional research of the SABATH gene in cucumbers.

## Figures and Tables

**Table 1:** List of 13 *CsSABATH* genes and basic characterizations.

Gene ID (v2)	Gene ID (v3)	Gene ID (v4)	Location	Protein Length (aa)	MW (Da)	PI	Instability Index	GRAVY Value	SC Localization
Csa3G002690	CsaV3_3G000680	CsaV4_3G000075	3(572314…574064)-	373	42131.04	6.81	51.15	−0.383	Cytoplasmic
Csa3G859730	CsaV3_3G045390	CsaV4_3G004520	3(37062534…37064406)-	375	42186.99	5.92	49.29	−0.260	Outer Membrane
Csa4G046610	CsaV3_4G005540	CsaV4_4G000567	4(3683812…3688124)-	368	41851.27	5.58	46.42	−0.375	Cytoplasmic/Outer Membrane
Csa5G241140	CsaV3_5G011240	CsaV4_5G000942	5(7294796…7298875)-	376	42357.16	5.73	44.83	−0.178	Cytoplasmic
Csa5G241640	CsaV3_5G011260	CsaV4_5G000943	5(7318739…7325466)-	361	40766.49	5.92	40.29	−0.237	Cytoplasmic
Csa5G313340	CsaV3_5G020180	CsaV4_5G001567	5(14937232…14943388)+	459	51873.85	6.25	42.59	0.046	Cytoplasmic /Outer Membrane
Csa6G503370	CsaV3_6G046470	CsaV4_6G003494	6(27448277…27453505)-	370	41317.27	6.19	41.06	−0.118	Outer Membrane /Cytoplasmic/Extracellular
Csa6G503870	CsaV3_6G046490	CsaV4_6G003496	6(27459877…27461717)-	371	42799.93	6.67	36.34	−0.326	Outer Membrane /Extracellular
Csa6G503880	CsaV3_6G046500	CsaV4_6G003497	6(27467723…27469134)-	295	33405.65	8.19	37.09	−0.101	Cytoplasmic/Outer Membrane
Csa6G503880	CsaV3_6G046510	CsaV4_6G003498	6(27470946…27472820)-	363	41359.27	5.41	43.61	−0.170	Outer Membrane /Cytoplasmic
Csa6G504380	CsaV3_6G046520	CsaV4_6G003499	6(27482065…27484009)+	364	41326.46	5.36	48.81	−0.215	Cytoplasmic
Csa6G504400	CsaV3_6G046540	CsaV4_6G003501	6(27487071…27489858)+	312	35717.29	8.84	49.54	−0.253	Cytoplasmic/Outer Membrane
Csa7G081680	CsaV3_7G020980	CsaV4_7G001138	7(9311426…9315269)-	385	42441.04	5.56	42.91	−0.134	Cytoplasmic

Note: The databases used in the experiment were all Chinese long v3 gene databases.

## Data Availability

Data will be made available on request.

## Supplementary Materials

The following supporting information can be downloaded at: https://www.mdpi.com/article/10.3390/plants14121748/s1, Table S1: Motif annotation; Table S2: Cucumber SABATH primer.
