# Supplementary material for: Characterization and Expression Analysis of the SABATH Gene Family Under Abiotic Stresses in Cucumber (Cucumis sativus L.)
Source: Plants (Basel). 2025 Jun 7;14(12):1748. doi: 10.3390/plants14121748 (PMC12196540; doi:10.3390/plants14121748)
Supplement: Supplementary file 1 [file plants-14-01748-s001.zip › Table S1.pdf]

motif	Sequence	Width	Description
1	GPCFFNGVPGSFYGRLFPKKSLHFVHSSYSLHWLSQVPEGL	41	SAM dependent carboxyl methyltransferase (IPR005299)
2	TSPKSVVEAYYKQFQKDFSLFLKCRAEELVSGGRMVLTLLGRTSEDPTS	49	SAM dependent carboxyl methyltransferase (IPR005299)
3	WELLALALNDMVKEGLIEEEKVDSFNIPYYMPSPEEVKEEIEKEGSFIIN	50	SAM dependent carboxyl methyltransferase (IPR005299)
4	LHQNKPPEYQVFLNDLPSNDFNTIFRSLP	29	SAM dependent carboxyl methyltransferase (IPR005299)
5	PTSISIADLGCSSGPNTLMIISEJIKQIE	29	SAM dependent carboxyl methyltransferase (IPR005299)
6	YNVAKCIRAVAEPLLISHFGEAIMDELFIRYGKIV	35	SAM dependent carboxyl methyltransferase (IPR005299)
7	GDTSYAKNSLLQRKVISMAWPI	22	None predicted
8	MEVCKILHMN	10	None predicted
9	NKGNIFI	7	None predicted
10	RIELAEPATWLKENIDIREWINHIRAAMEGIFIQHF	36	None predicted
